# The impact of social media use on college students’ mental health: a narrative review

**DOI:** 10.3389/fpubh.2026.1811098

**Published:** 2026-05-18

**Authors:** Chao Li

**Affiliations:** Henan Medical University, Xinxiang, Henan, China

**Keywords:** college students, mental health, psychological intervention, social media, social media dependence

## Abstract

With the rapid development and popularization of mobile internet technology, social media has deeply embedded itself into the daily lives and social patterns of contemporary college students, exerting complex and profound effects on their mental health. This topic has become a research hotspot in psychology, education, and public health. This article aims to systematically review the current research status on the dual effects of social media use on college students’ mental health. The review first outlines the background of social media as an important social medium and its prevalence and significance among college students. It then focuses on analyzing the positive psychological effects revealed by existing studies on social media use, such as expanding social support networks and enhancing a sense of belonging, as well as its potential negative risks, particularly its association with anxiety, depression, fluctuations in self-esteem, changes in real-life social skills, and social media dependence, which are core indicators of mental health. By integrating the latest empirical research findings, this article explores the psychological mechanisms behind these effects and proposes targeted educational guidance and psychological intervention suggestions based on existing evidence gaps and practical issues. This review aims to provide a comprehensive understanding of the multidimensional and complex relationship between social media and college students’ mental health, offering theoretical references and practical bases for the development of mental health education in universities and the formulation of relevant clinical intervention strategies.

## 1 Introduction

The popularity of social media has profoundly changed the social patterns and information-acquisition methods of contemporary college students, impacting their mental health in multiple dimensions. Social media has evolved from a supplementary communication tool into a core platform for social interaction, identity exploration, and information acquisition (1). The college student population is at a critical stage of psychological development, and the transformation brought on by college life, including academic pressure, identity recognition, and the reconstruction of interpersonal relationships, makes them more susceptible to psychological difficulties. Social media use may affect their self-identity, interpersonal relationships, and emotion-regulation abilities (2, 3). Existing studies indicate that there is a complex relationship between social media use and college students’ mental health, with both positive and negative effects coexisting. On the one hand, social media platforms provide unprecedented opportunities for social interaction, emotional support, and access to mental health resources (4–6). Additionally, during the coronavirus disease 2019 (COVID-19) pandemic, social media became a key coping mechanism, helping students maintain social connections and regulate negative emotions when physical social interactions were limited (7). On the other hand, problematic or excessive use of social media is positively correlated with increased risks of anxiety, depression, social media use disorder, and academic burnout (8, 9). In recent years, the emergence of new social platforms and features has further complicated the social media environment and may have different impacts on mental health (10, 11).

Studying the impact of social media on college students’ mental health has significant theoretical and practical implications. Although there is already a substantial amount of research on the impact of social media on mental health, existing reviews often fail to integrate findings from different theoretical frameworks, leading to limited observed mechanisms. Furthermore, the role of individual differences in moderating the impact of social media has not been sufficiently integrated, hindering the development of personalized intervention measures. Additionally, while some intervention studies have shown positive results, there is a lack of systematic evaluations of intervention strategies that promote healthy social media use.

In light of this, this review systematically organizes the current research on social media use and college students’ mental health, aiming to comprehensively analyze the bidirectional relationship between social media use and mental health-related outcomes; elucidate the underlying psychological, neurobiological, and socio-psychological mechanisms; explore individual characteristic factors that influence these relationships; assess the effectiveness of psychological intervention strategies; and propose future research directions to provide scientific evidence for evidence-based intervention practices in the field of college mental health.

## 2 Literature search and screening strategy

This review employs a rigorous literature search strategy aimed at identifying relevant research literature on social media use and college students’ mental health. Although this review is narrative in nature, a structured approach is adopted to ensure methodological rigor in identifying, selecting, and synthesizing the literature. Given the heterogeneity of research designs, measurement indicators, and outcome measures in this field, a narrative synthesis approach is considered most appropriate. This method facilitates a comprehensive analysis of the key themes and research questions outlined in the introduction while focusing on the convergence and divergence of findings across studies.

### 2.1 Search strategy

We conducted a literature search across four major academic databases: PubMed, PsycINFO, Web of Science, and the China National Knowledge Infrastructure. The search strategy used both controlled vocabulary terms and natural language keywords to enhance sensitivity. The search terms included: (“social media” OR “social network” OR “Instagram” OR “Facebook” OR “Douyin” OR “Twitter” OR “YouTube” OR “Snapchat” OR “WeChat” OR “Weibo”) AND (“college students*” (the asterisk indicates a wildcard for term expansion) OR “undergraduates*” OR “higher education”) AND (“mental health” OR “well-being” OR “depression” OR “anxiety” OR “stress” OR “psychological distress”). The asterisk (*) was used as a wildcard to expand search terms where applicable. This search strategy aimed to achieve as comprehensive coverage of the relevant literature as possible, thereby ensuring that the search results possess satisfactory breadth and accuracy.

The search was limited to peer-reviewed literature published in English or Chinese from January 2015 to December 2025. This time window was chosen to capture the evolution of social media platforms while focusing on user behavior patterns and their impact on specific aspects of mental health (such as anxiety and depression). Additionally, relevant reference lists of included studies and related review articles were manually screened to identify other eligible studies.

### 2.2 Inclusion and exclusion criteria

Studies were considered eligible for inclusion if they met the following criteria: first, the study population consisted of college students aged 18–25 years; second, rigorous quantitative, qualitative, or mixed-methods designs were employed; third, studies were published in peer-reviewed journals; and fourth, substantial insights were provided regarding the specific mechanisms, moderating factors, or intervention measures of social media use’s impact on mental health.

Conversely, studies were excluded if they focused solely on non-college-aged adolescents or adult populations, examined the impact of specific content without addressing broader social media usage patterns, lacked empirical data, or had questionable methodological quality.

### 2.3 Results of the search and selection

The initial literature search identified 1,491 records from PubMed, 472 records from PsycINFO, 693 records from Web of Science, and 972 records from CNKI, generating a total of 3,628 records across all electronic databases. An additional 6 records were identified through screening reference lists. After removal of duplicates and screening of titles, abstracts, and full texts, 92 studies ultimately met the inclusion criteria and were included in this review.

## 3 Positive effects of social media on college students’ mental health

### 3.1 Enhancing social support systems

Social media provides college students with a social support system that transcends temporal and spatial limitations, becoming an important supplement to maintaining real-life social relationships. Research indicates that, through instant messaging tools and social platforms, college students can maintain continuous contact with family and friends, effectively alleviating social isolation caused by spatial distance (12). A cross-sectional study among 1,257 college students found that social media may become a key channel for maintaining interpersonal relationships, helping users overcome feelings of loneliness brought about by social distancing policies (7). Social media not only sustains existing relationships but also facilitates new connections based on shared interests, academic pursuits, or identity characteristics. Online communities built around academic fields, extracurricular activities, or identity groups provide a platform for students to cultivate a sense of belonging. Wang et al.’s (13) study of 536 participants found that active participation through social media may effectively enhances the psychological resilience of Chinese college students, promoting both the seeking of social support and the enhancement of bidirectional communication with peers.

### 3.2 Providing emotional support and validation

Social media platforms offer important channels for emotional expression and seeking support, particularly for students who may be reluctant to disclose mental health issues in face-to-face interactions. The anonymity advantage of online mental health communities and the lower social stigma allow students to share personal struggles, seek advice, and gain validation from those with similar experiences. Social media provides diverse channels for emotional and informational support. Users can express emotional needs through various formats such as text, voice, and video, receiving immediate feedback. Social media helps to enhance subjective well-being, life satisfaction, and levels of social support, with college students’ participation in social networks contributing to the consolidation of existing friendships and the establishment of new connections through online platforms (14). Video-based platforms have become particularly effective channels for mental health support. Research by Choi et al. (10) indicates that videos showcasing personal narratives of mental health struggles on YouTube can provide social support, experiential validation, and foster help-seeking behaviors among young viewers. Similarly, social media influencers who establish genuine relationships with fans and share their personal mental health may journeys have been shown to effectively support college students’ mental health (11).

### 3.3 Promoting identity exploration and self-expression

The college stage is a critical period for identity formation, involving the exploration of personal values, beliefs, career aspirations, and social roles. Social media platforms provide unique opportunities for identity exploration and self-expression, which may be limited in real life. Research indicates that self-expression through social media is positively correlated with college students’ well-being, suggesting that these platforms are important tools for identity construction and self-discovery (15). The multimodal characteristics of contemporary social media allow for diverse self-expression, accommodating different dimensions of identity recognition. For special groups such as LGBTQ+, social media holds unique supportive value. These platforms provide relatively anonymous safe spaces for marginalized groups to find like-minded communities, gain identity recognition, and receive social support. Studies show that LGBTQ+ youth who establish online support networks through social media significantly reduce their levels of depression and anxiety (16). This “virtual sanctuary” function is particularly important in the absence of traditional social support systems, helping special groups to establish positive self-identities (17).

### 3.4 Access to mental health information and resources

Social media is increasingly becoming a primary channel for college students to access mental health information, including convenient, relevant, and timely content on mental health topics. During the COVID-19 pandemic, students who used social media to obtain health information exhibited greater health awareness (18, 19). Professional mental health accounts on social platforms also provide users with convenient access to mental health knowledge and self-help resources, potentially lowering the threshold for seeking help (20). The algorithmic personalization of social media content can accurately push mental health-related content to students who may benefit but lack proactive seeking awareness. Integrating mental health resources into the social platforms that students use daily can serve as an important means for conducting mental health literacy education, eliminating social stigma, and crisis intervention (21, 22).

## 4 Negative effects and risks of social media on college students’ mental health

### 4.1 Social media addiction and problematic use

Despite the benefits offered by social media, the excessive use of and addiction to it have become core issues affecting college students’ mental health. This dependency behavior is characterized by increased usage duration and a series of psychological and behavioral traits similar to those associated with substance addiction. Long-term, excessive social media use can trigger typical addiction features, including withdrawal symptoms and tolerance (23, 24). Research indicates that social media addiction is associated with high rates of anxiety, depression, stress, and academic burnout. Depression occupies a central position in the network linking social media addiction, academic burnout, and psychological distress, suggesting that depressive symptoms may serve as a key bridge connecting problematic social media use with broader functional impairments (9). Ahuja et al. (25) reported an 8.64% addiction rate among medical students in Punjab, India, with public university students being more susceptible than those in private institutions. A study involving 1,456 Chinese college students found a prevalence rate of 13.9% for depressive symptoms, with social appearance anxiety, body-checking behaviors, and social media addiction significantly positively correlated with depressive symptoms, indicating that social media addiction is an important risk factor for depressive symptoms among college students (26). Furthermore, social appearance anxiety and body-checking behaviors play independent and continuous mediating roles in the association between social media addiction and depressive symptoms (27). Students who develop psychological dependence on social media expend their attention resources on virtual interactions, leading to decreased academic engagement and concentration, thereby affecting academic performance and achievement.

### 4.2 Social comparison and fear of missing out (FoMO)

Social comparison is a key mechanism that triggers depression and anxiety. Social media content is carefully curated, with users primarily sharing positive experiences and achievements while neglecting mundane or negative aspects of life. This environment easily leads to upward social comparisons, resulting in decreased self-esteem, feelings of worthlessness, depression, and generalized anxiety (28, 29). This comparison, which is based on incomplete information, is a significant source of psychological distress in the social media environment. The impact of upward social comparison on mental health has been well-studied. The FoMO phenomenon, referring to a pervasive anxiety stemming from the fear of missing out on beneficial experiences of others, is prevalent among college students (30, 31) and can drive them to continuously monitor social media dynamics to maintain connections with their social circles. This persistent online presence and attention to others’ lives further exacerbates the frequency and duration of social media use, creating a vicious cycle. Tang and He noted that FoMO is a key mediating variable between social media addiction and academic procrastination, indicating that cognitive and emotional distress related to FoMO diverts college students’ attention and engagement from academic tasks (32).

### 4.3 Cyberbullying and negative social interactions

Social media can facilitate positive social interactions, but it also gives rise to negative interactions such as cyberbullying, harassment, and exclusion. A qualitative research indicates that exposure to false information may provoke anger or even depressive emotions among college students (33). Direct forms of negative social interactions, such as cyberbullying, have been shown to have a sustained association with adverse mental health outcomes. The post-traumatic stress responses resulting from cyberbullying should not be overlooked, as victims report higher rates of depression, anxiety, suicidal ideation, and self-harm behaviors (34). Studies indicate that adolescents who experience cyberbullying face a significantly increased risk of post-traumatic stress symptoms, including intrusive memories, avoidance behaviors, negative changes in cognition and emotion, and hypervigilance (35). Additionally, anonymous social platforms are associated with the depth of self-disclosure and the risk of cyberbullying; anonymity may encourage some users to share their true thoughts and emotional struggles while simultaneously weakening behavioral constraints among others, increasing the likelihood of online attacks and bullying (36).

### 4.4 Sleep disorders and circadian rhythm disruptions

The relationship between social media use and sleep is another key pathway through which it affects mental health. The timing patterns of social media use are closely related to sleep and mental health. Research by Bergaoui et al. (1) found that 94% of college students use social media before bedtime. Importantly, this habitual usage pattern may affect sleep hygiene, thereby harming mental health. Many college students are accustomed to using social media before sleep, and the blue light emitted by screens can inhibit melatonin secretion, disrupting the sleep–wake cycle. Additionally, receiving information or engaging in social interactions at night may trigger emotional fluctuations and cognitive arousal, leading to difficulties in falling asleep, reduced sleep duration, and decreased sleep quality (37). Ahuja et al. (25) noted that sleep deprivation caused by social media leads to distractions, increased risks of non-communicable diseases, and even adverse impacts on academic performance. An intervention study found a clear association between social media use and decreased sleep quality and circadian rhythm disruption, with participants experiencing significant relief from insomnia symptoms after a week of social media abstinence (38).

### 4.5 Information overload and cognitive overload

The vast amount of information on social media platforms can lead to cognitive overload, especially when students struggle to assess the credibility and relevance of content. Research indicates that misinformation on social media can provoke psychological distress, including confusion, frustration, and helplessness (33). Coping with cognitive load in a complex information environment may deplete psychological resources and lead to mental fatigue. Furthermore, the phenomenon of “doomscrolling,” characterized by compulsively scrolling through negative news and content, combines information-seeking with the reinforcement of negative emotions, potentially creating a cycle of anxiety and compulsive scrolling that is difficult to break (39). The algorithmic recommendation mechanisms of social media content expose users to extreme or anxiety-inducing content, further amplifying these negative effects.

## 5 Factors influencing the impact of social media on college students’ mental health

### 5.1 Cognitive behavioral mechanisms

Cognitive behavioral theory posits that individuals’ emotions and behaviors are not directly caused by events themselves, but rather by their cognitive appraisals, interpretations, and evaluations of those events. It provides a theoretical framework for understanding how thoughts, emotions, and behaviors interact to influence mental health. When applied to social media use, this theory emphasizes the role of cognitive processes such as attention allocation, interpretive biases, and rumination. The cognitive–behavioral mechanisms through which social media impacts college students’ mental health primarily involve the formation of irrational cognitions, the reinforcement of behavioral patterns, and the diversion of attention resources. First, irrational cognitions, particularly the pressure of presenting a perfect self, are one of the core mechanisms. College students tend to shape an idealized self-image to present on social media through careful selection and modification of content, driven by excessive concern for others’ evaluations and conditional self-worth (40). This cognitive pattern is closely related to social anxiety, depression, and other psychological distress (41). Second, The reinforcement mechanisms of social media (such as likes, comments, and shares) can create specific behavioral patterns, operating through a variable ratio schedule similar to gambling, which may foster habitual or compulsive usage patterns (42), thereby forming behavioral dependencies or even addictions (43). Additionally, the multitasking nature of social media can impair sustained attention, working memory, and executive functions; frequent switching between different applications, chat windows, and information streams can damage individuals’ sustained attention and cognitive control abilities (2, 44). Social media use consumes cognitive resources necessary for initiating and maintaining learning; thus, its cognitive load may have particularly significant impacts on learning activities (45). Prolonged exposure to high-intensity information flows and fragmented content may erode college students’ abilities for deep reading, critical thinking, and complex problem-solving, further impacting their sense of academic achievement and self-efficacy (46).

### 5.2 Psychosocial factors

According to social comparison theory, social media provides college students with unprecedented opportunities for upward social comparison, which is one of the significant mechanisms leading to mental health issues. This theory initially proposed by Leon Festinger, posits that in the absence of objective standards, individuals evaluate their own opinions, abilities, and attributes by comparing themselves with others. Depending on the direction of comparison (upward or downward), this process may trigger either positive or negative emotions and subsequently influence individuals’ self-evaluation and behavioral choices. College students are continuously exposed to idealized representations of peers’ achievements, social lives, and appearances on social media, which can lead to cognitive biases and trigger feelings of relative deprivation, envy, and jealousy (27, 47, 48). Upward comparisons based on academic achievements may also distort individuals’ learning motivations, shifting them from intrinsic interest-driven to externally performance-driven and competitive, thereby exacerbating psychological pressure and generating intense academic anxiety, fear of uncertainty about the future, and self-doubt, which can cumulatively lead to symptoms of depression and anxiety (49–51). Saha et al. (52) noted that the pressure to maintain an online image consistent with social expectations may induce significant anxiety, especially when students feel they must hide certain aspects of their true selves to conform to perceived norms.

Social support theory posits that the various forms of assistance and resources, including emotional, informational, and instrumental support, that individuals obtain through their social networks (e.g., family, friends, colleagues) can effectively buffer stress and promote physical and mental well-being. Perceived social support has been shown to be associated with better psychological outcomes. Social media platforms can enhance social support by facilitating connections with friends, family, and supportive communities, especially during times of stress or social isolation (13). However, the quality of online social support may differ from offline support, and excessive reliance on digital connections may increase feelings of loneliness if it replaces face-to-face interactions (53–55).

### 5.3 Differences in usage patterns and motivations

Uses and gratifications theory posits that audiences are not passive recipients of media content; instead, they actively select and use specific media to satisfy particular needs, including information acquisition, entertainment, social interaction, identity formation, based on their own social and psychological motivations. It shifts the analytical focus from the effects of media on individuals to how people use media. This theory emphasizes the agency of users in selecting and using media to satisfy specific needs and desires. The distinction between active and passive social media use is one of the key moderating factors of psychological effects (56). Active use (such as content creation and purposeful information acquisition) tends to satisfy individuals’ three basic psychological needs, and is associated with positive psychological effects, including enhanced self-efficacy and social connectedness (57). Passive use (referring to browsing others’ content without active participation) is more commonly linked to negative outcomes, including heightened social comparison, jealousy, and feelings of loneliness (58). This difference may arise because active use satisfies needs for self-expression, social connection, and competence, while passive use primarily activates social comparison and FoMO (59). The motivations for social media use also influence its psychological effects. Instrumental use (such as information retrieval, academic collaboration, or professional networking) driven by specific goals tends to yield more positive outcomes compared to entertainment use (characterized by passive content consumption and time-wasting) (60). Online social media can enhance the mental health of university students in Bangladesh and mediate the relationship between social media addiction and academic performance (61).

### 5.4 Individual differences and moderating factors

Personality traits play an important moderating role in the relationship between social media use and mental health. Studies have shown that personality traits have a positive direct effect on mental health and also exert positive effects on mental health through face-to-face communication-mediated social capital (62). Different personality traits shape distinct patterns of social media use motivation and behavior: agreeableness is a significant predictor of motivations to use social media, and all personality traits depict a moderate to strong relationship with information seeking (63). Regarding problematic use, as social media addiction scores increased, agreeableness, conscientiousness, and openness to improvement personality trait scores also increased (64). Furthermore, conscientiousness has the greatest effect on online information-seeking strategies, while agreeableness and neuroticism are related to several dimensions of social media-specific beliefs (65). Being female, introverted, conscientious, agreeable, and neurotic are associated with problematic social media use (66). Personality traits moderate the pathway through which social media use affects mental health by influencing individuals’ use motives, addiction propensity, and information-seeking strategies.

Self-esteem, as a key self-evaluation factor, influences the social comparison effects of social media. Individuals with low self-esteem are more susceptible to the negative psychological impacts of upward social comparisons, internalizing these comparisons as evidence of their inadequacies, leading to decreased self-worth and exacerbation of depressive symptoms (67–69). Conversely, individuals with high self-esteem exhibit more flexible coping strategies, being more likely to engage in downward comparisons (comparing with “less fortunate” individuals) or reinterpret comparison information to maintain their self-worth (70, 71).

### 5.5 Neurobiological mechanisms

From a neurobiological perspective, social media interactions activate the brain’s reward circuits, particularly the mesolimbic dopamine system projecting from the ventral tegmental area to the nucleus accumbens. This pathway is activated when social rewards such as likes, positive feedback, or social recognition are received (72, 73). Haddad et al. (4) noted that the activation of this reward system can trigger neuroadaptive changes similar to those seen in individuals with substance dependence, including tolerance (the need for increased stimulus intensity to achieve the same effect) and withdrawal responses (negative emotional states that occur when contact is limited). The college student population is particularly susceptible to the potential addictive effects of social media platforms due to the ongoing maturation of the prefrontal cortex, responsible for executive control and decision-making, and the developmental imbalance present between an overactive reward system and an immature prefrontal control system (74). Negative social experiences on social media, such as cyberbullying, social exclusion, or exposure to distressing content, can activate the hypothalamic–pituitary–adrenal axis, triggering a stress response characterized by increased cortisol secretion. Vornholt and De Choudhury (5) pointed out that the prolonged activation of the stress response system may lead to allostatic load and increase the risk of depression and anxiety. The relationship between social media use and stress may be bidirectional, with stress arising from problematic social media use and exacerbating such use.

### 5.6 Cultural background factors

The cultural background and social environment also moderate the relationship between social media use and mental health (75). Cultural values influence the types of normative social media behaviors and the psychological significance of social comparison processes (76, 77). Additionally, the specific functions and characteristics of different social media platforms may interact with individual traits, producing differentiated effects. To elucidate these complex interactions, longitudinal and cross-cultural studies are needed.

## 6 Intervention strategies for promoting healthy social media use

In today’s society, social media has been deeply integrated into the lives of college students, and literature highlights its associated risks for mental health issues, making the formulation of effective intervention strategies an urgent need in the public health field in recent years. Many promising intervention methods have been identified, but evidence of their effectiveness remains in preliminary stages and requires further research validation.

Intervention strategies grounded in psychological protective mechanisms may ameliorate mental health issues associated with social media use. A mediation analysis involving 579 university students revealed that social support mediates the associations among problematic social media use (PSMU), depressive symptoms, and life satisfaction, thereby effectively mitigating the impact of PSMU on students’ mental health (78). Another study, employing questionnaire-based methodologies and multivariate statistical analysis, provided important evidence for the design and implementation of interventions aimed at enhancing self-esteem and promoting positive childhood experiences to reduce the adverse effects of problematic social media use on loneliness (79). Furthermore, research has indicated that loneliness serves as a driver of PSMU, with resilience and meaning in life playing partial mediating roles. Consequently, interventions targeting loneliness and fostering resilience and life goals may help reduce the negative consequences associated with problematic social media use (80).

Internet-based cognitive behavioral therapy (iCBT) targeting problematic social media use significantly ameliorates depressive symptoms, anxiety symptoms, and social anxiety disorder among university students (81–84). Mindfulness-based interventions emphasize non-judgmental awareness of present experiences and have been shown to positively impact social media use issues and their psychological consequences (85, 86). Mindfulness interventions are suitable for addressing the automated and habitual characteristics of social media checking. By cultivating metacognitive awareness, students can gain deeper insights into their usage patterns and the psychological states that trigger problematic use (87, 88).

Research by Saha et al. (52) indicates that social media data can predict campus mental health service needs with high accuracy (86%), suggesting that monitoring online discussions can help optimize resource allocation and proactively initiate interventions. Furthermore, a cross-sectional online survey study conducted in Bangladesh suggests that when formulating policies and therapeutic interventions, priority should be given to examining social media usage patterns and behaviors. It also recommends rigorous scrutiny, timely updates, or strengthened protective measures regarding the security and privacy settings of social media platforms to reduce the risk of hazardous self-disclosure (89). Another cross-sectional study targeting university students in South Africa found a negative significant relationship between outcome expectations and external locus of control, and a positive significant relationship between external locus of control and mental health. This study suggested that governments should establish regulations to encourage social media practitioners to use localized content, thereby ensuring that citizens obtain appropriate and culturally relevant information through social media (90).

## 7 Limitations and recommendations

Existing literature and the methodology of this review impart several inherent methodological limitations. First, the field is predominantly characterized by cross-sectional studies, which limit our conclusions about the causal relationships between social media use and mental health outcomes. Second, measurement methods for social media use vary across studies: some studies employ self-report scales that may be influenced by recall bias and social desirability effects, while others use objective measurement tools that may not fully capture the complexity of usage patterns. Due to the heterogeneity of measurement indicators and outcomes across studies, a formal meta-analysis could not be conducted, leading to the adoption of a narrative synthesis approach in this work instead. Finally, the generalizability of research findings may be influenced by cultural factors, and it is therefore recommended that future research in this field continue in the following directions.

### 7.1 Longitudinal and multi-method research

First, future research should adopt longitudinal study designs to gain a deeper understanding of the long-term and dynamic impacts of social media use on college students’ mental health. Studies have confirmed that the negative impacts of social media use on mental health may take up to 18 months of follow-up observation to manifest (2). Shorter-term studies may therefore not capture these delayed effects, highlighting the importance of longitudinal research. Employing multi-method research that combines self-reports and behavioral and physiological objective measurement approaches can help uncover data biases and enhance measurement validity, providing a more comprehensive understanding of the impact of social media on mental health. Experimental designs that adjust social media exposure or test intervention strategies can provide stronger evidence for causal relationships. Randomized controlled trials of interventions, natural experiments arising from platform changes or policy implementations, and ecological momentary intervention studies providing real-time support are all promising methodological avenues.

### 7.2 Theoretical integration and cultural considerations

While existing studies have used different theoretical frameworks to analyze the impacts of social media, integrating more theoretical perspectives would significantly enhance explanatory power. Additionally, current research has overly focused on specific social groups, making it difficult to generalize findings to different cultural contexts. Fatima et al. (91) emphasize the need for cross-cultural research to understand how cultural factors moderate the impacts of social media. Cultural values, social norms, and platform ecosystems may all influence the relationship between social media use and mental health. Future research should include diverse cultural samples and explicitly validate hypotheses regarding cultural moderation effects.

### 7.3 Emerging technologies and positive technology research

Finally, as social media continues to evolve, the impacts of emerging social forms and technological applications on college students’ mental health need to be explored. The metaverse, as an immersive digital space integrating virtual reality, augmented reality, and other technologies, is reshaping social interactions, and the mental health impacts of metaverse socializing remain an underexplored area. The effectiveness, safety, and ethical issues of generative artificial intelligence–driven chatbots still require careful evaluation. The selective exposure effects of algorithmic recommendations create a new psychological environment, necessitating exploration of how the logic of algorithmic recommendations influences dynamic changes in emotional states, along with the assessment of the mental health benefits of interventions aimed at breaking the “information bubble.”

## 8 Conclusion

This review organizes the current research evidence on the complex relationship between social media use and college students’ mental health, deeply analyzing relevant mechanisms, moderating factors, and intervention strategies. The impact of social media on college students’ mental health is bidirectional and multifaceted (Supplementary Table 1). It is neither inherently beneficial nor harmful, but rather depends on the intricate interplay among individual characteristics, usage patterns, and platform affordances. Research indicates that social media can expand social support networks, provide emotional support, facilitate identity exploration, and offer convenient access to mental health resources. Active, goal-oriented use is more likely to yield positive psychological outcomes. However, passive browsing, excessive entertainment-oriented use, and compulsive use tend to precipitate addiction, upward social comparison, fear of missing out (FoMO), sleep disturbances, and exposure to cyberbullying, thereby significantly increasing the risk of negative emotional states such as anxiety and depression (Supplementary Table 2).

As social media continues to evolve, emerging technologies—including virtual reality (VR), artificial intelligence (AI), and augmented reality (AR)—are reshaping patterns of social interaction. Fundamental human needs for social connection, self-expression, competence enhancement, and autonomous decision-making are likely to continue shaping how college students engage with digital technologies and the subsequent effects on their mental health. Future research should employ longitudinal and experimental designs to establish causal directions, strengthen interdisciplinary theoretical integration and cross-cultural validation, and develop tailored intervention programs.

From a public health perspective, cultivating healthy digital living habits should not be viewed as a rejection of technology but rather as a pursuit of optimized technology use. Establishing conscious usage patterns, cultivating offline social skills, and developing diverse coping strategies for stress are essential. Universities should incorporate digital health literacy into core curricula to help students maintain a balanced state of mental health while enjoying the conveniences of technology. Through scientific research and collaboration among multiple stakeholders, the mental health development of the college student population can be promoted.
