# Supplementary material for: The impact of social media use on college students’ mental health: a narrative review
Source: Front Public Health. 2026 May 18;14:1811098. doi: 10.3389/fpubh.2026.1811098 (PMC13223138; doi:10.3389/fpubh.2026.1811098)
Supplement: Supplementary file 1 [file Data_Sheet_1.docx]

**Supplementary Table 1 Impact of Social Media on College Students’ Mental Health**

| **Type of Impact** | **Specific Argument** | **Representative Study (Author, Year)** | **Key Findings** |
| --- | --- | --- | --- |
| ****Positive effects**** | Enhancing social support systems | Wang et al., 2025 | Active social media use enhances psychological resilience and promotes social support seeking and peer communication among Chinese college students. |
|  | Providing emotional support and validation | Choi et al., 2021 | YouTube videos showing personal mental health struggles provide social support, experiential validation, and encourage help‑seeking behaviors in young viewers. |
|  | Promoting identity exploration and self‑expression | Sahoo et al., 2024 | Self‑expression through social media is positively correlated with well‑being; these platforms serve as important tools for identity construction and self‑discovery. |
|  | Access to mental health information and resources | Sharif et al., 2021 | During the COVID‑19 pandemic, students who used social media to obtain health information exhibited greater health awareness. |
| **Negative effects** | Social media addiction and problematic use | Lei et al., 2022 | Social media disorder is highly associated with anxiety, depression, stress, and academic burnout; depressive symptoms are a key bridge linking problematic use to functional impairment. |
|  |  | Ahuja et al., 2025 | Social media addiction rate among medical students in Punjab, India was 8.64%; public university students were more susceptible than those in private institutions. |
|  |  | Wang et al., 2025 | Social appearance anxiety and body checking behaviors play independent and serial mediating roles between social media addiction and depressive symptoms. |
|  | Social comparison and Fear of Missing Out (FoMO) | Ulvi et al., 2022 | Carefully curated positive content on social media easily leads to upward social comparison, resulting in decreased self‑esteem, feelings of worthlessness, depression, and generalized anxiety. |
|  |  | Tang & He, 2025 | FoMO is a key mediator between social media addiction and academic procrastination; related cognitive and emotional distress diverts attention from academic tasks. |
|  | Cyberbullying and negative social interactions | Hinduja & Patchin, 2025 | Cyberbullying is consistently associated with depression, anxiety, suicidal ideation, and self‑harm behaviors; victims report significant post‑traumatic stress responses. |
|  |  | Sachser et al., 2025 | Adolescents experiencing cyberbullying have significantly increased risk of post‑traumatic stress symptoms, including intrusive memories, avoidance, negative alterations in cognition/mood, and hypervigilance. |
|  | Sleep disorders and circadian rhythm disruptions | Bergaoui et al., 2025 | 94% of college students use social media before bedtime; this habitual pattern affects sleep hygiene and thus harms mental health. |
|  |  | Calvert et al., 2025 | A clear association was found between social media use and decreased sleep quality and circadian rhythm disruption; participants showed significant relief from insomnia after one week of abstinence. |
|  | Information overload and cognitive overload | Jiang & Wang, 2024 | Misinformation on social media provokes psychological distress, including confusion, frustration, and helplessness. |
|  |  | Saritepeci et al., 2022 | “Doomscrolling” (compulsively scrolling through negative news) combines information‑seeking with negative emotion reinforcement, creating a difficult‑to‑break cycle of anxiety and compulsive scrolling. |

**Supplementary Table 2 Factors Influencing the Impact of Social Media on College Students’ Mental Health**

| **Category** | **Specific Mechanism / Factor** | **Representative Study (Author, Year)** | **Key Findings** |
| --- | --- | --- | --- |
| ****Cognitive‑behavioral mechanisms**** | Irrational cognitions | Tóth et al., 2022 | Irrational beliefs and perfectionism are closely related to social anxiety, depression, and other psychological distress. |
|  | Behavioral reinforcement | Sun et al., 2024 | Information sharing characteristics under recommendation algorithms are associated with problematic behaviors among college students. |
|  | Attention resource fragmentation and multitasking | Sohail et al., 2025 | Frequent switching between different applications and information streams impairs sustained attention and cognitive control. |
| ****Psychosocial factors**** | Upward social comparison | Irmer & Schmiedek, 2023 | The association between daily social media use and well‑being is mediated by upward comparisons. |
|  |  | Xu & Li, 2024 | Upward social comparison influences social anxiety through the chain mediation of relative deprivation and rumination. |
|  | Social support | Wang et al., 2025 | Perceived social support is associated with better psychological outcomes; social media can enhance social support, especially during stress or social isolation. |
|  |  | Stone et al., 2022 | Over‑reliance on digital connections may replace face‑to‑face interactions and increase loneliness. |
| ****Usage patterns and motivations**** | Active use vs. passive use | Mao & Zhang, 2023 | Active use (content creation, purposeful information seeking) is associated with positive outcomes; passive use (browsing others’ content) is more linked to negative outcomes. |
|  | Instrumental vs. entertainment use | Wu et al., 2024 | Instrumental use (information retrieval, academic collaboration) yields more positive psychological results than entertainment‑oriented passive consumption. |
| ****Individual differences**** | Personality traits | Kircaburun et al., 2020 | Being female, introverted, conscientious, agreeable, and neurotic are associated with problematic social media use. |
|  |  | Mercan & Uysal, 2023 | As social media addiction scores increased, scores for agreeableness, conscientiousness, and openness to experience also increased. |
|  | Self‑esteem | Samra et al., 2022 | Individuals with low self‑esteem are more susceptible to the negative psychological impacts of upward social comparison, leading to decreased self‑worth and exacerbated depressive symptoms. |
|  |  | Wang, 2024 | A longitudinal relationship exists between self‑esteem and problematic social media use. |
| ****Neurobiological mechanisms**** | Brain reward circuit (mesolimbic dopamine system) | Kang et al., 2023 | Social rewards (likes, positive feedback) activate projections from the ventral tegmental area to the nucleus accumbens, potentially triggering neuroadaptive changes similar to substance dependence. |
|  | HPA axis stress response | Vornholt & De Choudhury, 2021 | Negative social experiences (e.g., cyberbullying, social exclusion) activate the HPA axis, increasing cortisol secretion; prolonged activation raises the risk of depression and anxiety. |
| ****Cultural background factors**** | Cultural values and social norms | Wang et al., 2025 | Cultural values influence normative social media behaviors and the psychological meaning of social comparison processes. |
|  |  | Zhang et al., 2025 | The impact of English usage on social media on interpersonal communication intentions is mediated by language confidence and English cultural identity. |
